# Supplementary material for: COZOID: contact zone identifier for visual analysis of protein-protein interactions
Source: BMC Bioinformatics. 2018 Apr 6;19:125. doi: 10.1186/s12859-018-2113-6 (PMC5889581; doi:10.1186/s12859-018-2113-6)
Supplement: Supplementary file 2 — Software build. Executable binary file of the software tool. (ZIP 118,784 kb) [file 12859_2018_2113_MOESM2_ESM.zip › cozoid/java/docs/beansbinding-1.2.1-doc.zip!/index-files/index-9.html]

I-Index (Beans Binding)


---


|  |  |  |
| --- | --- | --- |
| **Overview** \| Package \| Class \| Use \| **Tree** \| **Deprecated** \| **Index** \| **Help** | |  |
| **PREV LETTER**   **NEXT LETTER** | **FRAMES**    **NO FRAMES**     **All Classes** |  |


A B C D E F G H I J K L M N O P Q R S T U V W 

---


## **I**

**id** - Variable in class org.jdesktop.el.impl.parser.SimpleNode: **Identifier()** - Method in class org.jdesktop.el.impl.parser.ELParser: **IDENTIFIER** - Static variable in interface org.jdesktop.el.impl.parser.ELParserConstants: **ILLEGAL\_CHARACTER** - Static variable in interface org.jdesktop.el.impl.parser.ELParserConstants: **image** - Variable in class org.jdesktop.el.impl.parser.SimpleNode: **image** - Variable in class org.jdesktop.el.impl.parser.Token: The string image of the token. **IMPL\_OBJ\_START** - Static variable in interface org.jdesktop.el.impl.parser.ELParserConstants: **IN\_EXPRESSION** - Static variable in interface org.jdesktop.el.impl.parser.ELParserConstants: **inBuf** - Variable in class org.jdesktop.el.impl.parser.SimpleCharStream: **input\_stream** - Variable in class org.jdesktop.el.impl.parser.ELParserTokenManager: **inputStream** - Variable in class org.jdesktop.el.impl.parser.SimpleCharStream: **INSTANCEOF** - Static variable in interface org.jdesktop.el.impl.parser.ELParserConstants: **Integer()** - Method in class org.jdesktop.el.impl.parser.ELParser: **INTEGER\_LITERAL** - Static variable in interface org.jdesktop.el.impl.parser.ELParserConstants: **invoke(ELContext, Object[])** - Method in class org.jdesktop.el.impl.MethodExpressionImpl: Evaluates the expression relative to the provided context, invokes the method that was found using the supplied parameters, and returns the result of the method invocation. **invoke(ELContext, Object[])** - Method in class org.jdesktop.el.impl.MethodExpressionLiteral: **invoke(EvaluationContext, Class[], Object[])** - Method in class org.jdesktop.el.impl.parser.AstIdentifier: **invoke(EvaluationContext, Class[], Object[])** - Method in class org.jdesktop.el.impl.parser.AstValue: **invoke(EvaluationContext, Class[], Object[])** - Method in interface org.jdesktop.el.impl.parser.Node: **invoke(EvaluationContext, Class[], Object[])** - Method in class org.jdesktop.el.impl.parser.SimpleNode: **invoke(ELContext, Object[])** - Method in class org.jdesktop.el.MethodExpression: If a String literal is specified as the expression, returns the String literal coerced to the expected return type of the method signature. **isBigDecimalOp(Object, Object)** - Static method in class org.jdesktop.el.impl.lang.ELSupport: **isBigIntegerOp(Object, Object)** - Static method in class org.jdesktop.el.impl.lang.ELSupport: **isBound()** - Method in class org.jdesktop.beansbinding.Binding: Returns whether or not this `Binding` is bound. **isDoubleOp(Object, Object)** - Static method in class org.jdesktop.el.impl.lang.ELSupport: **isDoubleStringOp(Object, Object)** - Static method in class org.jdesktop.el.impl.lang.ELSupport: **isEditable()** - Method in class org.jdesktop.swingbinding.JTableBinding.ColumnBinding: Returns whether or not the cells of the column should be editable. **isEditable()** - Method in class org.jdesktop.swingbinding.JTableBinding: Returns whether or not the cells of the table should be editable. **isListening(S)** - Method in class org.jdesktop.beansbinding.PropertyHelper: Returns whether or not there are any `PropertyStateListeners` installed for the given source object. **isListening()** - Method in class org.jdesktop.swingbinding.adapters.BeanAdapterBase: **isLiteralText()** - Method in class org.jdesktop.el.Expression: Returns whether this expression was created from only literal text. **isLiteralText()** - Method in class org.jdesktop.el.impl.MethodExpressionImpl: **isLiteralText()** - Method in class org.jdesktop.el.impl.MethodExpressionLiteral: **isLiteralText()** - Method in class org.jdesktop.el.impl.ValueExpressionImpl: **isLiteralText()** - Method in class org.jdesktop.el.impl.ValueExpressionLiteral: **isLongOp(Object, Object)** - Static method in class org.jdesktop.el.impl.lang.ELSupport: **isManaged()** - Method in class org.jdesktop.beansbinding.Binding: Returns whether or not this `Binding` is managed. **isNumber(Object)** - Static method in class org.jdesktop.el.impl.lang.ELArithmetic: **isNumberType(Class)** - Static method in class org.jdesktop.el.impl.lang.ELArithmetic: **isPropertyResolved()** - Method in class org.jdesktop.el.ELContext: Returns whether an `ELResolver` has successfully resolved a given (base, property) pair. **isPropertyResolved()** - Method in class org.jdesktop.el.impl.lang.EvaluationContext: **isReadable(S)** - Method in class org.jdesktop.beansbinding.BeanProperty: Returns whether or not the `Property` is readable for the given source. **isReadable(S)** - Method in class org.jdesktop.beansbinding.ELProperty: Returns whether or not the `Property` is readable for the given source. **isReadable(Object)** - Method in class org.jdesktop.beansbinding.ObjectProperty: Returns `true`; `ObjectProperty` is always readable. **isReadable(S)** - Method in class org.jdesktop.beansbinding.Property: Returns whether or not the `Property` is readable for the given source. **isReadable(S)** - Method in class org.jdesktop.beansbinding.PropertyHelper: Returns whether or not the `Property` is readable for the given source. **isReadable()** - Method in class org.jdesktop.beansbinding.PropertyStateEvent: Returns whether or not the `Property` is currently readable for the source object. **isReadOnly(ELContext, Object, Object)** - Method in class org.jdesktop.el.ArrayELResolver: If the base object is a Java language array, returns whether a call to `ArrayELResolver.setValue(org.jdesktop.el.ELContext, java.lang.Object, java.lang.Object, java.lang.Object)` will always fail. **isReadOnly()** - Method in class org.jdesktop.el.BeanELResolver.BeanProperty: **isReadOnly(ELContext, Object, Object)** - Method in class org.jdesktop.el.BeanELResolver: If the base object is not `null`, returns whether a call to `BeanELResolver.setValue(org.jdesktop.el.ELContext, java.lang.Object, java.lang.Object, java.lang.Object)` will always fail. **isReadOnly(ELContext, Object, Object)** - Method in class org.jdesktop.el.CompositeELResolver: For a given `base` and `property`, attempts to determine whether a call to `CompositeELResolver.setValue(org.jdesktop.el.ELContext, java.lang.Object, java.lang.Object, java.lang.Object)` will always fail. **isReadOnly(ELContext, Object, Object)** - Method in class org.jdesktop.el.ELResolver: For a given `base` and `property`, attempts to determine whether a call to `ELResolver.setValue(org.jdesktop.el.ELContext, java.lang.Object, java.lang.Object, java.lang.Object)` will always fail. **isReadOnly(EvaluationContext)** - Method in class org.jdesktop.el.impl.parser.AstDeferredExpression: **isReadOnly(EvaluationContext)** - Method in class org.jdesktop.el.impl.parser.AstDynamicExpression: **isReadOnly(EvaluationContext)** - Method in class org.jdesktop.el.impl.parser.AstIdentifier: **isReadOnly(EvaluationContext)** - Method in class org.jdesktop.el.impl.parser.AstValue: **isReadOnly(EvaluationContext)** - Method in interface org.jdesktop.el.impl.parser.Node: **isReadOnly(EvaluationContext)** - Method in class org.jdesktop.el.impl.parser.SimpleNode: **isReadOnly(ELContext)** - Method in class org.jdesktop.el.impl.ValueExpressionImpl: **isReadOnly(ELContext)** - Method in class org.jdesktop.el.impl.ValueExpressionLiteral: **isReadOnly(ELContext, Object, Object)** - Method in class org.jdesktop.el.ListELResolver: If the base object is a list, returns whether a call to `ListELResolver.setValue(org.jdesktop.el.ELContext, java.lang.Object, java.lang.Object, java.lang.Object)` will always fail. **isReadOnly(ELContext, Object, Object)** - Method in class org.jdesktop.el.MapELResolver: If the base object is a map, returns whether a call to `MapELResolver.setValue(org.jdesktop.el.ELContext, java.lang.Object, java.lang.Object, java.lang.Object)` will always fail. **isReadOnly(ELContext, Object, Object)** - Method in class org.jdesktop.el.ResourceBundleELResolver: If the base object is not null and an instanceof `ResourceBundle`, return `true`. **isReadOnly(ELContext)** - Method in class org.jdesktop.el.ValueExpression: Evaluates the expression relative to the provided context, and returns `true` if a call to `ValueExpression.setValue(org.jdesktop.el.ELContext, java.lang.Object)` will always fail. **isSelected()** - Method in class org.jdesktop.swingbinding.adapters.AbstractButtonAdapterProvider.Adapter: **isSourceUnreadableValueSet()** - Method in class org.jdesktop.beansbinding.Binding: Returns the value of the `sourceUnreadableValueSet` property, which indicates whether or not the `sourceUnreadableValue` property is set on the `Binding`. **isStringFloat(String)** - Static method in class org.jdesktop.el.impl.lang.ELSupport: **isWriteable(S)** - Method in class org.jdesktop.beansbinding.BeanProperty: Returns whether or not the `Property` is writeable for the given source. **isWriteable(S)** - Method in class org.jdesktop.beansbinding.ELProperty: Returns whether or not the `Property` is writeable for the given source. **isWriteable(Object)** - Method in class org.jdesktop.beansbinding.ObjectProperty: Returns `false`; `ObjectProperty` is never writeable. **isWriteable(S)** - Method in class org.jdesktop.beansbinding.Property: Returns whether or not the `Property` is writeable for the given source. **isWriteable(S)** - Method in class org.jdesktop.beansbinding.PropertyHelper: Returns whether or not the `Property` is writeable for the given source. **isWriteable()** - Method in class org.jdesktop.beansbinding.PropertyStateEvent: Returns whether or not the `Property` is currently writeable for the source object.

---


|  |  |  |
| --- | --- | --- |
| **Overview** \| Package \| Class \| Use \| **Tree** \| **Deprecated** \| **Index** \| **Help** | |  |
| **PREV LETTER**   **NEXT LETTER** | **FRAMES**    **NO FRAMES**     **All Classes** |  |


A B C D E F G H I J K L M N O P Q R S T U V W 

---
